# Stemming the tide of distrust: A mixed-methods study of vaccine hesitancy

**DOI:** 10.1017/cts.2022.492

**Published:** 2022-11-09

**Authors:** Andrew Plunk, Brynn Sheehan, Shelly Orr, Danielle Gartner, F. Gerard Moeller, Praduman Jain

**Affiliations:** 1 Department of Pediatrics, Eastern Virginia Medical School, Norfolk, VA, USA; 2 Department of Psychiatry and Behavioral Sciences, Eastern Virginia Medical School, Norfolk, VA, USA; 3 Healthcare Analytics and Delivery Science Institute, Eastern Virginia Medical School, Norfolk, VA, USA; 4 Virginia Commonwealth University Health System, Richmond, VA, USA; 5 Vibrent Health, Fairfax, VA, USA; 6 Wright Center for Clinical and Translational Research, Virginia Commonwealth University, Richmond, VA, USA

**Keywords:** COVID-19, vaccine hesitancy, participant trust, pandemic response, research participation, CBPR

## Abstract

Public distrust in the US pandemic response has significantly hindered its effectiveness. In this community-based participatory research mixed-methods study, based on two datasets, we examined how distrust in COVID-19 vaccines relates to institutional distrust. We found that the Johnson & Johnson vaccine pause undermined trust in COVID-19 vaccines in general. Findings also suggest that vaccine distrust developed after participating in a study on COVID-19 testing. Increased distrust may be an unintended consequence of how healthcare and public health activities are presented and delivered, and research participation is structured. Both will continue without proactively addressing the root causes of distrust.

## Introduction

Lack of public trust has significantly undermined the effectiveness of the COVID-19 pandemic public health response. Unfortunately, a longstanding history of institutional distrust has affected the perceived trustworthiness of specific aspects of the response, such as vaccination [1].

The aim of this study was to examine how distrust in COVID-19 vaccination might occur as an unintended consequence of other institutional practices for which distrust is longstanding. To explore this conception and capture information from disparate perspectives, we combined data from two sources. The first source used a community-based participatory research approach to gather qualitative and quantitative perspectives on institutional distrust and adherence to public health guidance. For this, we worked with a cohort of low-income housing residents in several cities in Southeastern and Central Virginia. The second source of data sources were collected during a clinical research project focused on in-home COVID-19 testing in Richmond, Virginia. The combination of these data sources was coordinated by the Wright Center for Clinical and Translational Research, Virginia Commonwealth University’s (VCU) Clinical Translational Science Award (CTSA)-funded center. This approach combined targeted community engagement with analyses based on a broader sampling strategy.

## Methods

Two sources of data were employed. They are described separately below.

### COVIDCARE Study

Participants 18 years or older were recruited through advertisements on Virginia Commonwealth University’s (VCU) website, email lists, and flyers on the VCU campus. Participants joined the study through a secure study website where they provided electronic consent and were instructed through the study tasks. Research staff were available by phone and in-person to answer participants’ questions. All data were collected between June and October 2021, when Delta was the dominant SARS-CoV-2 variant in the USA.

Participants were asked to complete two QuickVue at-home COVID-19 tests, an in-clinic PCR test in the Richmond, VA area, and a series of electronic surveys throughout the study. Participants completed these activities on their own schedule, using their own electronic devices. Average time of participation was 1–10 days.

Compensation was pro-rated based on completed study tasks, with a maximum compensation of $175 via electronic gift card. The project was approved by Western Institutional Review Board, IRB (WIRB; study 1309332). The data collection effort was approved by VCU’s IRB (HM20022035) and deferred to WIRB. Analysis of deidentified data was approved by George Mason University IRB (1743684-1).

### Housing Collaborative Sample

The research team at Eastern Virginia Medical School (EVMS) has conducted multiple waves of data collection in partnership with residents of low-income housing in several cities in Southeastern and Central Virginia. Eligibility criteria were being an adult resident of low-income housing in the Virginia cities of Chesapeake, Hampton, Newport News, Norfolk, Portsmouth, Richmond, Suffolk, or Virginia Beach. Recruitment was conducted using flyers, recontact based on participation in previous studies, and referral from the community advisory board (CAB) and other participants.

In June 2021, recruitment began for a cohort that would participate in ongoing research activities (N = 187). The present study involved data collected during 71 individual semi-structured interviews from April 28 through June 1, 2021; 24 focus group discussions (N = 102 participants) from June 11, 2021 through January 12, 2022, and multiple waves of survey assessment from May 4 through November 29, 2021 (N = 111 participants). As compensation, participants were given unlimited internet connectivity via provided tablets and $5 for each completed research activity, equaling an upper range of $400. Approval was obtained from the EVMS IRB (20-04-NH-0099, 21-03-EX-0069, and 21-03-FB-0046).

### Qualitative Methods

The Housing Collaborative qualitative analyses presented here is part of a larger effort to develop a grounded theory [2] of low-income housing resident distrust in COVID-related public health guidance. In the current work, our findings are primarily exploratory, having served to develop context and informed subsequent quantitative assessment development. A CAB comprised of low-income housing residents was consulted to develop goals for the interviews and focus group discussions and create a list of guiding questions. CAB members were full partners during this process; the initial list of guiding questions was developed over the course of several meetings. The CAB also provided feedback on subsequent drafts of an interview guide and approved a final draft before submission to the IRB. Additional details are available in the Supplemental Material.

### Quantitative Outcome Measures and Covariates

Our primary outcomes in analyses of the Housing Collaborative sample came from responses to several items assessing trust in institutions involved with the COVID-19 pandemic response and attitudes about the Johnson & Johnson Janssen (J&J) COVID-19 vaccine pause from April 13, 2021 through April 23, 2021 [3]. The main outcome variable in analysis of the COVIDCARE sample was an indicator for developing distrust in COVID-19 vaccines during participation in the study. Covariates for all analyses included gender, race/ethnicity (White, Black/African American, American Indian/Alaska Native, Asian, other racial identity, or any Hispanic Ethnicity), high school educational attainment, and age category (18–29, 30–39, 40–49, 50–59, 60–69, and 70 and older). These variables are explained in greater detail below and in the Supplemental Material.

### Quantitative Statistical Analysis Plan

Analyses based on the Housing Collaborative sample used *t* tests to explore bivariate differences in institutional and vaccine-related trust constructs based on vaccination status and bivariate and multivariate linear regression to estimate associations between institutional trust and reactions to the J&J pause with trust in COVID-19 vaccine efficacy. Version 4.1.0 of R was used for analysis. In the COVIDCARE sample, logistic regression was used to assess the odds of developing vaccine distrust during participation in the project. Additional details are available in the Supplemental Material.

## Results

### Sample Descriptions

Demographic characteristics by sample and vaccination status are displayed in Table 1. The COVIDCARE sample was more racially diverse, younger, more highly educated, and contained a larger proportion of vaccinated participants. The proportion of female participants was similar across samples. While the overall distribution was quite different, educational attainment among the unvaccinated was relatively similar between samples. The proportion of vaccinated Black participants was also similar (67% vs. 74%; percentage not depicted in Table 1).

**Table 1.** Demographic characteristics by vaccination status

	Housing Collaborative Sample			COVIDCARE		
	Total No. (%)	Vaccinated No. (%)	Unvaccinated No. (%)	Total No. (%)	Vaccinated No. (%)	Unvaccinated No. (%)
Total	111 (100)	73 (66)	38 (34)	634 (100)	521 (82)	113 (18)
* Race/ethnicity*						
Non-Hispanic White	5 (5)	2 (3)	3 (7)	314 (49)	271 (52)	37 (33)
Non-Hispanic Black/African American	103 (93)	69 (95)	34 (88)	280 (44)	206 (40)	72 (64)
Non-Hispanic AI/AN	--	--	--	9 (1)	9 (2)	--
Non-Hispanic Asian	--	--	--	21 (3)	19 (4)	2 (2)
Any Hispanic ethnicity	--	--	--	15 (3)	14 (3)	1 (1)
Other	4 (2)	2 (2)	2 (5)	10 (2)	3 (1)	1 (1)
* Gender*						
Man/male	12 (11)	11 (18)	1 (2)	197 (31)	158 (30)	39 (35)
Woman/female	82 (74)	52 (67)	30 (70)	431 (68)	357 (69)	74 (65)
Non-binary	--	--	--	5 (1)	5 (1)	--
Transgender	--	--	--	1 (1)	1 (1)	--
None of these describe me	15 (14)	9 (14)	6 (26)	--	--	--
Prefer not to say	2 (2)	1 (1)	1 (2)	--	--	--
* Age*						
18–29	10 (9)	2 (3)	8 (21)	186 (29)	149 (29)	37 (33)
30–39	22 (20)	8 (11)	14 (37)	150 (24)	119 (23)	31 (27)
40–49	12 (11)	6 (8)	6 (16)	129 (20)	105 (20)	24 (21)
50–59	23 (21)	18 (25)	5 (13)	96 (15)	81 (16)	15 (13)
60–69	29 (26)	25 (34)	4 (11)	60 (9)	55 (11)	5 (4)
70+	15 (14)	14 (19)	1 (3)	13 (2)	12 (2)	1 (1)
* Educational attainment*						
No high school	24 (22)	18 (24)	6 (15)	12 (2)	7 (1)	5 (4)
High school	28 (25)	21 (29)	7 (24)	93 (15)	62 (12)	31 (27)
Some college	36 (32)	19 (24)	17 (41)	182 (29)	133 (26)	49 (43)
College degree	23 (21)	15 (22)	8 (20)	347 (55)	319 (61)	28 (25)

Percentages are based on subgroup and may not total 100% due to rounding. Abbreviation: AI/AN American Indian/Alaska Native.

### Housing Collaborative Interview and Focus Group Feedback About Institutional Trust and Distrust in Vaccines

Participants described how information about the pandemic was received by marginalized communities in the context of their experiences before the pandemic. In particular, distrust of government and the public health establishment is ubiquitous, even among those willing to become vaccinated. Notably, the “government” label is broadly applied. In an interview with a 38-year-old Black female participant, she explained:

Participant: *Um, the way that African Americans have been treated for years, things that we went through. The first thing, we have no separation with the government, the police department, any of that. In my community’s eyes, it’s all one. So, whatever, however they’ve been tainted, it is then through arrest in the community, if it’s been from eviction from public housing, however their life has been tainted from a public agency, it just continued on*.

Interviewer: *Do you think that extends to public health workers?*

Participant: *Yes, ma’am. They feel as if they work hand in hand together. Like I said, our public health officials, our police department, social service, they feel as if it’s all one family*.

The removal of the J&J COVID-19 vaccine from the market in April 2021 was identified by participants as an important source of distrust. As one 65-year-old Black female participant stated, “With everything that’s gone on in our history, it was hard for us to do any of this and you all took us for granted again. We’re just guinea pigs. Again. We feel betrayed.” Resentment due to feeling experimented upon has since been a common reaction from our participants in response to other news. For example, in response to learning about emergency use authorizations after the full approval of the Pfizer-BioNTech COVID-19 Vaccine, a 32-year-old Black male participant states: “I’ve been in drug trials before, but not anymore. We were just guinea pigs this whole time, before the vaccine got approved. What were they doing, giving it to us before it was fully approved?”

### Housing Collaborative Surveys on Institutional Trust and Distrust in Vaccines

Bivariate differences in institutional trust and perceptions of the J&J vaccine pause by vaccination status are reported in Table 2. While distrust in the J&J vaccine and White elected officials was high for both groups, responses to other items exhibited significant differences by vaccination status.

**Table 2.** Trust items by vaccination status

	Vaccinated		Unvaccinated		*t*(110)	*p*
	M	SD	M	SD		
Generally speaking, how much do you trust the federal government in Washington?	3.13	1.06	4.05	0.97	4.75	<.001
I trust that the coronavirus vaccines are effective.	2.00	0.77	3.42	1.17	7.03	<.001
Pausing the use of the Johnson and Johnson vaccine caused me to trust it less.	3.66	1.23	3.61	1.40	−0.19	.85
The Johnson and Johnson vaccine pause has changed how I feel about the other vaccines.	2.42	1.21	3.13	1.45	2.65	.009
*How much do you feel you can trust the role each of these has played in the coronavirus vaccination effort?*						
Your usual doctor or healthcare team	1.66	0.88	2.52	1.21	3.89	<.001
The U.S. Food and Drug Administration (FDA)	2.43	1.02	3.14	1.20	3.04	.004
Drug companies who worked to create and test the vaccines	2.76	1.04	3.68	0.94	4.66	<.001
Scientists who worked to create and test the vaccines	2.43	0.99	3.47	1.00	5.21	<.001
Pharmacies and walk-in clinics where people can get vaccinated	2.07	0.93	3.36	1.19	5.84	<.001
Black religious leaders in your community	2.60	1.02	3.24	1.32	2.46	.02
Black community organizers in your area	2.52	0.96	3.11	1.23	2.47	.02
Black elected officials in your community	2.66	1.01	3.35	1.23	2.85	<.001
White elected officials in your community	3.27	1.13	3.71	1.20	1.77	.08

Comparisons are *t* tests with unadjusted p-values. All items were scored on a 5-point scale indicating either agreement/disagreement with the statement or the level of trust held (completely, mostly, somewhat, not much, not at all). Lower values indicate greater trust. M = mean, SD = standard deviation.

We categorized 8% of the sample – 23% of unvaccinated individuals – as having been strongly influenced by the J&J pause (indicated by the endorsement of “agree” or “strongly agree” on both of the J&J pause items). Using bivariate regression, having been strongly influenced by the J&J pause was associated with 85% higher distrust in COVID-19 vaccine effectiveness relative to the rest of the sample (*b* = 1.42, *P* = 0.004). Further, having been strongly influenced by the J&J pause continued to be associated with higher distrust in COVID-19 vaccine effectiveness in a multivariable regression model controlling for demographic characteristics and the other trust variables. Overall, a strong reaction to the J&J pause was associated with 152% higher distrust in COVID-19 vaccines (*b* = 1.18, *P* = 0.04). Unvaccinated individuals exhibited 288% higher distrust in COVID-19 vaccines relative to their vaccinated peers, as indicated by a significant interaction term (*b* = 2.23, *P* = 0.007).

### The Association Between Trust in COVID-19 Vaccines and Participation in the COVIDCARE Study

Seventeen individuals indicated that they had developed distrust in COVID-19 vaccines between their initial assessment and follow-up, representing 15% of unvaccinated COVIDCARE participants. Of these, 82% (N = 14) identified as Black or African American. Using logistic regression, Black racial identity and failing to complete high school were associated with 552% and 783% higher odds, respectively, of reporting distrust in COVID-19 vaccination after having participated in the study (*OR* = 5.52, 95% CI [2.46, 8.57], *OR* = 7.83, 95% CI [3.97, 11.69]).

## Discussion

Qualitative and quantitative results from the Housing Collaborative sample show clear associations between institutional distrust and vaccination. Participants broadly reported perceptions of responsibility and feelings of trust (or distrust) across institutions. Notably, reaction to the J&J pause was associated with increased distrust in all COVID-19 vaccine efficacy, after controlling for existing institutional distrust. This may indicate increased susceptibility to information discrediting vaccines and suggests that perceived missteps in one area could elicit skepticism in others. This is particularly salient as it relates to the COVID-19 response effort. Testing, vaccinations, recommendations, and mandates—and the institutions that promote them—are not easily disentangled by the lay public.

Results from the COVIDCARE study further emphasized that the public health response to COVID-19 is seen as a uniform effort. An unexpected finding was that vaccine distrust developed throughout participation in the study among participants who had not previously indicated distrust in the vaccine, particularly among Black individuals who did not complete high school. By allowing for between-groups comparisons, these findings complement and expand upon those from the Housing Collaborative sample. Small sample sizes are a notable limitation of our study. We also made no attempt to produce broadly generalizable samples, although consistency of our findings across two very different samples speaks to their potential relevancy to other settings.

Distrust continues to be relevant in the public health response to COVID-19, and experiences in one area have the potential to impact others. Public health professionals, clinicians, researchers, and their institutions must acknowledge that they play a role in affecting individual trust and decision-making. Concerted efforts to become more trustworthy could include proactively addressing historical and contemporary inequalities that perpetuate lack of faith and skepticism. Without efforts to directly address these issues related to trust, unintended consequences related to the process of receiving healthcare or participating in research will likely continue.

## Supplementary material

For supplementary material accompanying this paper visit https://doi.org/10.1017/cts.2022.492. click here to view supplementary material
