# Supplementary material for: Stemming the tide of distrust: A mixed-methods study of vaccine hesitancy
Source: J Clin Transl Sci. 2022 Nov 9;6(1):e128. doi: 10.1017/cts.2022.492 (PMC9794956; doi:10.1017/cts.2022.492)
Supplement: Supplementary file 1 [file S2059866122004927sup001.docx]

**Supplemental Material**

**Community Engagement Methods.** The Eastern Virginia Medical School team (EVMS; ADP and BES) uses a community-based participatory research approach with laypeople as active participants in the research process.^1,2^ A community advisory board (CAB) comprised of low-income housing residents is our primary partner. The CAB was initially established in 2014 with membership from Norfolk, VA. The partnership has spanned several grant-funded projects, first focusing on respiratory health and childhood asthma, and later on studies examining smoke-free public housing.^3–5^ CAB members contribute to all stages of research, including dissemination. Monthly in-person meetings were held at the EVMS campus before the pandemic, with approximately 15 CAB members in regular attendance prior to March, 2020. We shifted to a virtual engagement approach as limitations to face-to-face contact were put into place. This included providing CAB members with tablets, unlimited data connectivity, and ongoing technical support. The CAB saw rapid growth with the development of this capacity. We transitioned to weekly meetings and were also able to expand to a regional presence, which currently includes residents from the Virginia cities of Chesapeake, Hampton, Portsmouth, Newport News, Richmond, Roanoke, Suffolk, and Virginia Beach, in addition to Norfolk. There are 28 current members, all of whom live in some form of low-income housing in one of these cities.^6^ For the current study, the CAB participated in activities involving the Housing Collaborative sample. This included focus group discussion guide development and interpretation of findings as they fit into the broad context of mistrust in the U.S. COVID-19 pandemic response. The CAB was not involved in activities related to the COVIDCARE Study or to efforts to link results from the two data sets (i.e., the Housing Collaborative Sample and the COVIDCARE Study sample).

**Qualitative Methods**

***Rapid Ethnography.*** Our initial 71 semi-structured interviews were based on a rapid ethnographic assessment approach.^7^ This approach produces timely data useful for connecting with marginalized or vulnerable populations that are difficult to reach. The Eastern Virginia Medical School (EVMS) team was part of a national consortium (CommuniVax) devoted to engaging historically underserved Black, Hispanic/Latino, and Indigenous populations in the U.S. A report designed for community dissemination summarizing early findings was produced.^5^

The discussion guide is available in Table S1. The interviews were conducted and analyzed using codes provided by the Commnivax consortium by masters-level research staff with formal training in qualitative methods.

***Grounded Theory.*** The focus group discussions in the Housing Collaborative study used a Grounded Theory (GT) approach with the aim of generating an explanatory theory of COVID-related decision making.^8^ The GT approach is based on the following key components:

*Openness.* GT emphasizes inductive reasoning (i.e., moving from the particular to the general). This requires an open approach to the process being studied.^9^ GT requires minimal preconception and researchers using this method must allow a GT study to evolve as the data dictate.^10^

*Immediate analysis.* Data analysis begins after the first interview and is an ongoing activity in a GT study.^9,10^ This allows for theoretical sampling (see below).

*Coding and comparing.* GT data analysis is based on line-by-line coding and comparison.^9,10^ This ensures that coding categories are “grounded” in the data. “Pet” themes and ideas of the researchers are also forced out unless they have an emergent fit.^10^

*Memo-writing.* This is the primary data-analytic activity in GT, based on reflecting on the coding process.^11^ Constant comparison during the memo-writing process helps dismiss preconceived notions and other “baggage,” while driving the analysis forward.^10^ Notably, GT data analysis is not software-driven; memos are constantly categorized and sorted by the researchers conducting the analysis.

*Theoretical sampling.* This activity is informed by coding, comparison and memo-writing.^9^ Theoretical sampling is the data collection process in which theory is developed as it emerges by deciding what data to collect next through a joint process of collecting, coding and analyzing data.^10^ The careful selection of participants and refinement of questions allows researchers to fill gaps, address uncertainty, and build an emerging theory.^9^

*Theoretical saturation.* In GT, saturation is not simply the point after which the researchers hear nothing new from participants. Instead, it means that all concepts in the theory are well-understood and substantiated by the data (i.e., the theory is “grounded” in the data).^9^ This is informed by “interchangeability” of emerging concepts, in which generating additional properties of a code in new data does not explain additional variation.^10^

*Production of a substantive theory.* The goal of GT is to develop a substantive theory featuring concepts that are related as a cohesive whole.^9^

***Focus Group Data Analysis Procedure.*** The following procedure was used to conduct and analyze our focus group and in-depth interviews.

*Initial research questions.* Initial interviews were initiated with simple “grand tour” questions that broadly introduced the topics and started the conversation. The goal was to have “guided conversations” that did not influence the scope or depth of participant responses.^12^ For the initial interviews, we wanted to indirectly assess attitudes toward COVID-19 testing and other guidance to ensure that we do not “preconceive the emergence of the data.”^13^

*Data analysis.* Memo-writing occurred after each interview and coding session. Coding was based on the transcribed interviews. Initial codes were refined and focused based on ongoing data analysis (i.e., constant comparisons). Analysis was conducted by a team of three individuals at EVMS: the study PI, a PhD-trained ethicist and epidemiologist with formal training in qualitative methods and several years of experience conducting qualitative research in low-income settings, a PhD-level health systems researcher with experience conducting qualitative process-improvement studies, and a master’s-level public health practitioner with multiple years of practical experience facilitating focus group discussions. This team met weekly via video teleconference to discuss the codes and to refine questions for subsequent interviews. The process of iterative question refinement over time is captured in Table S2. HyperRESEARCH version 4.5.2 (Researchware, Inc., Randolph, MA) was used for organizing the coding process. Results presented in the current study represent a portion of data analysis directly related to the Johnson and Johnson COVID-19 Vaccine pause.

**Quantitative Methods**

***Housing Collaborative Study Analysis Outcome Measures.*** Qualitative feedback informed the creation of questionnaire items asking respondents to rate their agreement with the J&J pause items listed in Table 2 of the main text. Other trust items in the questionnaire were drawn from the COVID collaborative survey.^14^ For the regression analyses (described below), the trust tems were converted into binomial indicators denoting “agreement” or “strong agreement” (for the J&J pause items) or “completely” or “mostly” level of trust in the other trust items.

***COVIDCARE Study Analysis Outcome Measures.*** The outcome variable for this analysis was based on an item assessing factors affecting vaccination decisions. Our “vaccine distrust” variable was coded as “0” or “1” based on a response during the follow-up assessment of “I do not trust the vaccine” when asked the question “What factors might make you less likely to get the vaccine?”

***Race/ethnicity and gender covariate coding.*** Racial and ethnic identity were assessed similarly in both studies, and have been recoded as any Hispanic ethnicity, non-Hispanic White, non-Hispanic Black, non-Hispanic American Indian/Alaska Native, non-Hispanic Asian, or other Non-Hispanic racial identity. Gender was assessed slightly differently between studies—the Housing Collaborative sample was asked how they identify with the following options: “female,” “male,” “none of these describe me,” and “prefer not to answer.” The COVIDCARE sample was asked “What terms best express how you describe your gender identity?” with the options “man,” “woman,” “non-binary,” “transgender,” “none of these describe me, and I'd like to consider additional options” and “prefer not to answer.”

***Regression Modeling.*** Version 4.1.0 of R was used for analysis. The Housing Collaborative analyses used multiple regression with an interaction term to allow the impact of the J&J pause and trust items to vary based on vaccination status. Estimates for two models, the first without and the second with all the trust items listed in Table 2, are available in Table S3 (the bivariate regression model described in the main text is not depicted). The COVIDCARE Study Analysis used logistic regression. Estimates for the model are available in Table S4.

**References for the Supplementary Material**

1. Clinical and Translational Science Awards Consortium Community Engagement Key Function Committee Task Force on the Principles of Community, ed. Executive Summary. In: *Principles of Community Engagement*. 2nd ed. U.S. Department of Health and Human Services; 2011:3-41.

2. Sanders Thompson VL, Ackermann N, Bauer KL, Bowen DJ, Goodman MS. Strategies of community engagement in research: definitions and classifications. *Transl Behav Med*. 2020;11(2):441-451. doi:https://doi.org/10.1093/tbm/ibaa042

3. Plunk AD, Rees VW, Jeng A, Wray JA, Grucza RA. Increases in Secondhand Smoke After Going Smoke-Free: An Assessment of the Impact of a Mandated Smoke-Free Housing Policy. *Nicotine Tob Res*. Published online 2020. doi:10.1093/ntr/ntaa040

4. Wray JA, Sheehan BE, Rees VW, Cooper D, Morgan E, Plunk AD. A Qualitative Study of Unfairness and Distrust in Smoke-free Housing. *Am J Health Behav*. 2021;45(5):798-809. doi:10.5993/AJHB.45.5.1

5. Plunk AD, Kiger PG, DiazGranados D, et al. *Addressing Hampton Roads Community Mistrust in the Wake of the Pandemic*. The Johns Hopkins Center for Health Security; 2021. Accessed January 15, 2021. https://www.centerforhealthsecurity.org/our-work/Center-projects/communivax/local-reports/211103-CommuniVax-Local-Report-VAHamptonRoads.pdf

6. Plunk AD, Carver A, Minggia C, et al. Virtual Engagement of Under-resourced Communities: Lessons Learned During the COVID-19 Pandemic for Creating Crisis-Resistant Research Infrastructure. *J Clin Transl Sci*. 2022;6(1):e44.

7. Sangaramoorthy T, Kroeger KA. *Rapid Ethnographic Assessments: A Practical Approach and Toolkit For Collaborative Community Research*. Routledge; 2020.

8. Glaser BG, Strauss AL. *The Discovery of Grounded Theory: Strategies for Qualitative Research*. Aldine de Gruyter; 1967. doi:10.4324/9780203793206

9. Sbaraini A, Carter SM, Evans RW, Blinkhorn A. How to do a grounded theory study: a worked example of a study of dental practices. *BMC Med Res Methodol*. 2011;11(1):128. doi:10.1186/1471-2288-11-128

10. Glaser BG, Holton J. Remodeling Grounded Theory. *Forum Qual Sozialforschung Forum Qual Soc Res*. 2004;5(2). doi:10.17169/fqs-5.2.607

11. Saldaña J. *The Coding Manual for Qualitative Researchers*. Sage; 2015.

12. Rubin HJ, Rubin IS. *Qualitative Interviewing: The Art of Hearing Data*. Sage; 2011.

13. Glaser BG. *Basics of Grounded Theory Analysis: Emergence vs Forcing*. Sociology press; 1992.

14. Sparks S, De Jong A, Filer C, Langer G. *COVID Collaborative Survey: Coronavirus Vaccination Hesitancy in the Black and Latinx Communities*.; 2020. Accessed March 23, 2022. https://www.covidcollaborative.us/assets/uploads/pdf/COVIDCollaborativeVaccinationAttitudesSurveyReport2.docx

| Table S2. Focus Group Questions | | | |
| --- | --- | --- | --- |
|  | Dates | No. Focus Groups | No. Participants |
| Initial Grand Tour Questions | 6/11/21 – 7/7/21 | 3 | 26 |
| 1. What do you think about wearing masks to help protect people from COVID-19? | | | |
| 1. What comes to mind when you think about COVID-19 tests? | | | |
| 1. What comes to mind when you think about COVID-19 vaccines? | | | |
|  |  |  |  |
| First revision | 7/14/21 – 7/20/21 | 2 | 13 |
| 1. What comes to mind when you think about COVID-19 vaccines?* | | | |
| 1. Can you all tell me what you've heard about population control and the COVID-19 vaccine? | | | |
| 1. Would any information or education that people provide address any concerns you may have? | | | |
| 1. Who do you trust to get information about COVID-19 from? | | | |
| 1. Do you think masks protect you from COVID-19?* | | | |
| 1. How comfortable are the masks? | | | |
| 1. What comes to mind when you think about COVID-19 tests?* | | | |
| 1. Do you trust the tests? | | | |
|  | | | |
| Second Revision | 7/28/21 – 8/18/21 | 4 | 22 |
| 1. What comes to mind when you think about COVID-19 vaccines? | | | |
| 1. Do you all remember when they took the J&J vaccine off of the market? How did that make you feel about vaccines? | | | |
| 1. Can you all tell me what you've heard about population control and the COVID-19 vaccine? | | | |
| 1. Would any information or education that people provide address any concerns you may have? | | | |
| 1. Who do you trust to get information about COVID-19 from? | | | |
| 1. Do you think masks protect you from COVID-19? | | | |
| 1. How comfortable are the masks? | | | |
| 1. What comes to mind when you think about COVID-19 tests? | | | |
| 1. Do you trust the tests? | | | |

| Table S1. In-depth Interview Questions |
| --- |
| 1. Considering everything going on in your life and with the pandemic, what are your biggest concerns? |
| 1. Tell me about your experience with COVID-19 so far. |
| 1. Will you get a COVID-19 vaccine? Why or why not? |
| 1. What do you need to be able to get vaccinated for COVID-19? |
| 1. What does your community need to recover from the pandemic and how can COVID-19 vaccination fit in? |

| Table S2. Focus Group Questions, Cont. | | | |
| --- | --- | --- | --- |
|  | Dates | No. Focus Groups | No. Participants |
| Third Revision | 8/27/21 – 9/29/21 | 5 | 24 |
| 1. What comes to mind when you think about COVID-19 vaccines? | | | |
| 1. Do you all remember when they took the J&J vaccine off of the market? How did that make you feel about vaccines? | | | |
| 1. How do you all feel about all the new information that's come out about the vaccines lately?    1. Follow-Up: How did you feel when they told everyone to start wearing masks again?    2. Follow-Up: How did you all feel when they announced that we might need booster shots of vaccines?    3. Follow-Up: How did you all feel when the Pfizer vaccine was fully approved? | | | |
| 1. Has your level of trust in the vaccines changed over the last few months? Why?    1. Follow-Up: For those of you that have gotten vaccinated, has this made you question whether or not you should have gotten the vaccine?    2. Follow-Up: Does it make you feel differently about booster shots? | | | |
| 1. Can you all tell me what you've heard about things like sterilization or killing specific groups of people and the COVID-19 vaccine? | | | |
| 1. Would any information or education that people provide address any concerns you may have? | | | |
| 1. Who do you trust to get information about COVID-19 from? | | | |
| 1. Why do you trust those sources of information? | | | |
| 1. Do you think masks protect you from COVID-19? | | | |
| 1. How comfortable are the masks? | | | |
| 1. What comes to mind when you think about COVID-19 tests? | | | |
| 1. Do you trust the tests? | | | |

| Table S2. Focus Group Questions, Cont. | | | |
| --- | --- | --- | --- |
|  | Dates | No. Focus Groups | No. Participants |
| Fourth Revision | 10/20/21 – 10/26/21 | 2 | 10 |
| 1. What makes you trust someone? | | | |
| 1. Who do you all trust? | | | |
| 1. Are you comfortable with what you know about the COVID-19 vaccines? Why?* | | | |
| 1. Do you all remember when they took the J&J vaccine off of the market? How did that make you feel about vaccines? | | | |
| 1. How does it make you all feel when you turn on the news and there’s a change in guidance?    1. Follow-Up: How did you feel when they told everyone to start wearing masks again?    2. Follow-Up: How did you all feel when they announced that we might need booster shots of vaccines?    3. How did you all feel when the Pfizer vaccine was fully approved? | | | |
| 1. Has your level of trust in vaccines changed over the last few months? Why?    1. Follow-Up: For those of you that have gotten vaccinated, has this made you question whether or not you should have gotten the vaccine?    2. Follow-Up: Does it make you feel differently about booster shots? | | | |
| 1. Can you all tell me what you've heard about things like sterilization or killing specific groups of people and the COVID-19 vaccine? | | | |
| 1. Would any information or education that people provide address any concerns you may have about COVID-19? | | | |
| 1. Who do you trust to get information about COVID-19 from? | | | |
| 1. Why do you trust those sources of information? | | | |
| 1. Do you think masks protect you from COVID-19? | | | |
| 1. Do you trust that the COVID-19 tests are giving good results? | | | |

| Table S2. Focus Group Questions, Cont. | | | |
| --- | --- | --- | --- |
|  | Dates | No. Focus Groups | No. Participants |
| Fifth Revision | 12/13/21 – 1/12/21 | 2 | 8 |
| 1. What makes you trust someone? | | | |
| 1. Who do you all trust? | | | |
| 1. Are you comfortable with what you know about the COVID-19 vaccines? Why? | | | |
| 1. Do you all remember when they took the J&J vaccine off of the market? How did that make you feel about vaccines? | | | |
| 1. How does it make you all feel when you turn on the news and there’s a change in guidance? Like mask guidance/boosters?    1. How did you feel when they told everyone to start wearing masks again?    2. How did you all feel when they announced that we might need booster shots of vaccines?    3. How did you all feel when the Pfizer vaccine was fully approved? | | | |
| 1. Has your level of trust in vaccines changed over the last few months? Why?    1. For those of you that have gotten vaccinated, has this made you question whether or not you should have gotten the vaccine?    2. Does it make you feel differently about booster shots? | | | |
| 1. Is anyone here less likely to get the flu shot this year than they were last year or the year before? [If so, could you explain why?]    1. What about for your children?    2. Has COVID impacted your trust in getting the flu shot for your children?    3. What about other childhood vaccinations (has your trust changed in these)? | | | |
| 1. Is anyone less likely to trust information from the local health department or a federal agency like the CDC or FDA than they were last year or the year before? | | | |
| 1. Who do you trust to get information about COVID-19 from? | | | |
| 1. Why do you trust those sources of information? | | | |
| 1. Have your experiences since the pandemic changed how you might feel about being in a drug trial? | | | |

| Table S3. Housing Collaborative Regression Analysis Predicting Distrust in COVID-19 Vaccine Effectiveness | | | | | | |
| --- | --- | --- | --- | --- | --- | --- |
|  | Model 1 | | | Model 2 | | |
|  | B | SE | P | B | SE | P |
| Intercept | 1.67 | 0.61 | 0.007 | 0.77 | 0.68 | 0.26 |
| High School Education | 0.16 | 0.21 | 0.47 | 0.37 | 0.27 | 0.18 |
| *Race/Ethnicity (ref = White)* |  |  |  |  |  |  |
| Black/African American | -0.23 | 0.41 | 0.57 | -0.74 | 0.42 | 0.08 |
| Other | -0.37 | 0.96 | 0.69 | -0.55 | 0.81 | 0.50 |
| *Age (ref = 18-29)* |  |  |  |  |  |  |
| 30-39 | 0.68 | 0.35 | 0.05 | 0.74 | 0.44 | 0.14 |
| 40-49 | 1.34 | 0.39 | < 0.001 | 1.19 | 0.46 | 0.02 |
| 50-59 | 0.25 | 0.37 | 0.50 | 0.67 | 0.44 | 0.14 |
| 60-69 | 0.61 | 0.36 | 0.09 | 0.79 | 0.40 | 0.06 |
| 70+ | 0.64 | 0.42 | 0.12 | 0.90 | 0.45 | 0.05 |
| *Gender (ref = male)* |  |  |  |  |  |  |
| Female | -0.25 | 0.28 | 0.37 | -0.25 | 0.30 | 0.41 |
| None of these describe me | -0.26 | 0.35 | 0.47 | 0.04 | 0.36 | 0.90 |
| Prefer not to say | 1.18 | 0.67 | 0.08 | 2.44 | 0.83 | 0.006 |
| Vaccinated | -1.31 | 0.21 | < 0.001 | -1.57 | 0.83 | 0.07 |
| Affected by the J&J pause | 1.42 | 0.62 | 0.03 | 1.18 | 0.55 | 0.04 |
| J&J x Vaccinated | -1.25 | 0.73 | 0.09 | -2.23 | 0.79 | 0.007 |
| *Trust* |  |  |  |  |  |  |
| Doctors |  |  |  | -0.05 | 0.16 | 0.74 |
| Scientists |  |  |  | 0.03 | 0.16 | 0.85 |
| Drug companies |  |  |  | 0.07 | 0.17 | 0.33 |
| Pharmacies and Clinics |  |  |  | 0.13 | 0.15 | 0.42 |
| FDA |  |  |  | 0.07 | 0.16 | 0.65 |
| Federal government |  |  |  | -0.07 | 0.12 | 0.58 |
| White Elected Officials |  |  |  | 0.02 | 0.14 | 0.84 |
| Black Elected Officials |  |  |  | -0.02 | 0.18 | 0.87 |
| Black Community Organizers |  |  |  | 0.18 | 0.29 | 0.52 |
| Black Religious Leaders |  |  |  | -0.08 | 0.26 | 0.77 |
| Doctors x Vaccinated |  |  |  | 0.24 | 0.29 | 0.42 |
| Scientists x Vaccinated |  |  |  | 0.25 | 0.26 | 0.34 |
| Drug companies x Vaccinated |  |  |  | 0.27 | 0.30 | 0.38 |
| Pharmacies and Clinics x Vaccinated |  |  |  | 0.16 | 0.27 | 0.84 |
| FDA x Vaccinated |  |  |  | 0.33 | 0.24 | 0.18 |
| Federal government x Vaccinated |  |  |  | -0.05 | 0.27 | 0.83 |
| White Elected Officials x Vaccinated |  |  |  | -0.02 | 0.30 | 0.95 |
| Black Elected Officials x Vaccinated |  |  |  | 0.18 | 0.36 | 0.63 |
| Black Community Organizers x Vaccinated |  |  |  | -0.13 | 0.58 | 0.81 |
| Black Religious Leaders x Vaccinated |  |  |  | 0.18 | 0.36 | 0.63 |

| Table S4. COVIDCARE Study Analysis Predicting Distrust in COVID-19 Vaccines | | | |
| --- | --- | --- | --- |
|  | B | SE | P |
| Intercept | -2.43 | 0.90 | 0.007 |
| Gender (*ref = man*) |  |  |  |
| Woman | -0.70 | 0.44 | 0.11 |
| Non-Binary | -6.40 | 7.30 | 0.99 |
| Transgender | -7.56 | 9.33 | 0.99 |
| *Race/ethnicity (ref = non-Hispanic white)* | | | |
| Non-Hispanic Black | 1.71 | 0.57 | 0.002 |
| Non-Hispanic AI/AN | -4.77 | 4.98 | 0.99 |
| Non-Hispanic Asian | -4.97 | 5.24 | 0.99 |
| Other non-Hispanic | -5.02 | 5.62 | 0.99 |
| Any Hispanic ethnicity | -5.71 | 6.35 | 0.99 |
| High School Education | -2.05 | 0.69 | 0.003 |
| *Age (ref = 18-29)* |  |  |  |
| 30-39 | -0.92 | 0.72 | 0.20 |
| 40-49 | -0.88 | 0.70 | 0.21 |
| 50-59 | -0.35 | 0.63 | 0.58 |
| 60-69 | -0.30 | 0.65 | 0.64 |
| 70+ | -7.10 | 23.45 | 0.99 |
| Vaccinated | -3.63 | 5.15 | 0.97 |
